# Paediatric Posttraumatic Cyst-Like Lesions: A Report of Two Cases and Review of the Literature

**DOI:** 10.1155/2020/7834969

**Published:** 2020-04-15

**Authors:** R. Jansen, F. van Rooijen, J. P. Toirkens, A. T. Besselaar, J. J. Tolk

**Affiliations:** ^1^Máxima MC, Department of Orthopaedic Surgery and Trauma, P.O. Box 90052, 5600 PD Eindhoven, Netherlands; ^2^Máxima MC, Department of Radiology, P.O. Box 90052, 5600 PD Eindhoven, Netherlands

## Abstract

Posttraumatic cyst-like lesions are a rare finding after greenstick fractures in children. These asymptomatic, cystic lesions become radiologically apparent 2-4 months after the initial trauma. Conventional radiographs typically show nonexpansile, well-circumscribed lesions in the cortex, proximal to the fracture site. It is important to recognize them as benign to prevent unnecessary concern and additional imaging or invasive diagnostic procedures. No treatment is indicated, as they eventually resolve spontaneously in 1 to 3 years. The two cases of posttraumatic cyst-like lesions after paediatric forearm fractures are presented.

## 1. Introduction

Posttraumatic cyst-like lesions in children are quite a rare phenomenon with only approximately 30 cases reported in literature [1–5]. The cystic lesions are usually first spotted on conventional radiographs as nonexpansile, well-circumscribed lucent lesions in the cortex, no larger than 10 mm [4, 6]. In more than 80% of the cases, the lesions were located in the distal radius. They become radiologically apparent 2-4 months and occur most frequently after greenstick or torus fracture of the distal radius in children [1]. These are benign lesions and should not be confused with more aggressive or expansive lesions such as osteomyelitis or bone tumours [1–6]. It is therefore important to be informed about these benign posttraumatic bone cysts to prevent unnecessary further diagnostics or even invasive diagnostic methods.

## 2. Case

### 2.1. Case 1

**Figure 1:**
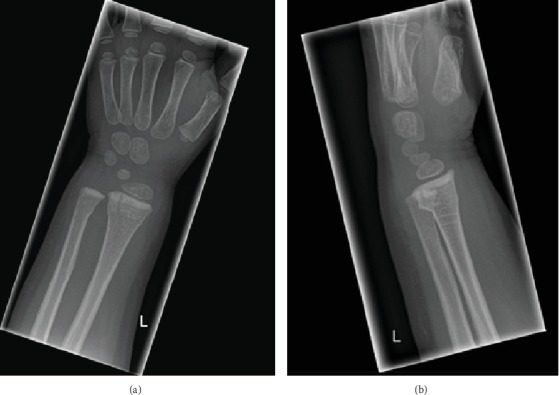
(a, b) Anteroposterior and lateral radiographs of the left wrist showing Salter-Harris type 2 fracture of the distal radius, with minimal dorsal displacement and a small line on the ulna suggestive for an ulnar fissure.

A 4-year-old female presented with pain over the left wrist after a fall down the stairs. Clinical examination revealed tenderness over the distal radius and ulna with a limited range of motion. Initial X-rays showed a Salter-Harris type 2 fracture of the distal radius, with minimal dorsal displacement and a small line on the ulna suggestive for an ulnar fissure (Figures 1(a) and 1(b)).

**Figure 2:**
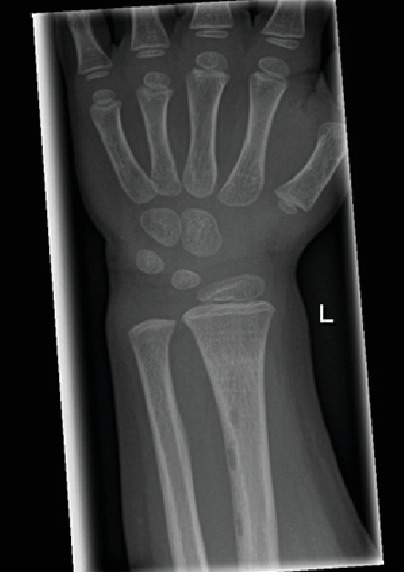
Anteroposterior radiograph showing complete consolidation and three well-circumscribed lucent lesions in the cortex, proximal to the previous fracture site.

She was treated conservatively with a below-elbow cast and regained a full range of motion after only two weeks of treatment. After six months follow-up, an X-ray was obtained to monitor potential growth disturbance. The images showed the fracture had healed completely and showed no signs of growth disturbance. However, it also revealed the three well-circumscribed lucent lesions in the cortex, proximal to the earlier fracture site (Figure 2). The cysts were asymptomatic with no signs of inflammation, osteolysis, or signs suggesting osteomyelitis and were therefore recognized as benign posttraumatic bone cysts. At one-year follow-up, no additional lesions or growth of the lesions was discovered. No further follow-up or investigation was deemed necessary.

### 2.2. Case 2

**Figure 3:**
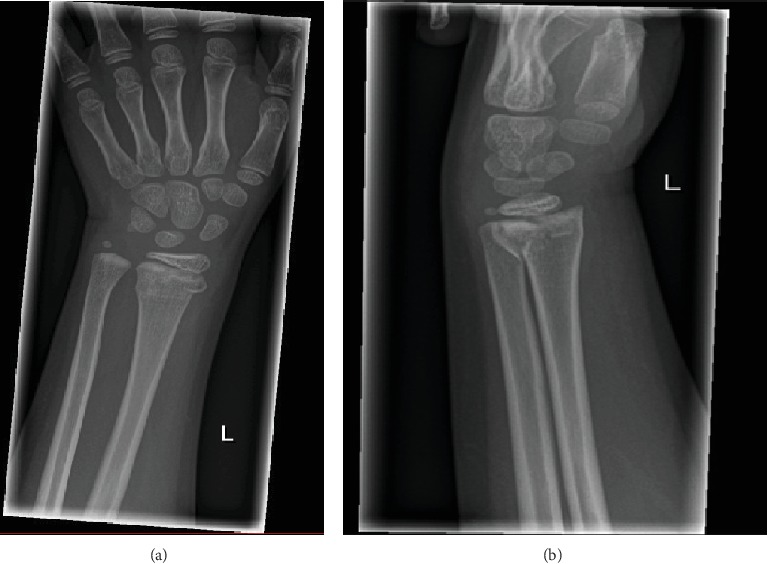
(a, b) Anteroposterior and lateral radiographs of the left wrist distal forearm fracture showing a Salter-Harris type 2 fracture of the distal radius and a greenstick fracture of the distal ulna.

A 6-year-old girl presented with a painful and swollen left wrist after a fall. Clinical examination showed tenderness and soft tissue swelling over the distal radius with no limitations in range of motion. X-rays showed a distal forearm fracture with a Salter-Harris type 2 fracture of the distal radius and a greenstick fracture of the distal ulna (Figures 3(a) and 3(b)). She was treated conservatively with a below-elbow cast for four weeks. At 4 weeks check-up, there was a good clinical recovery and X-rays showed a normal healing process.

**Figure 4:**
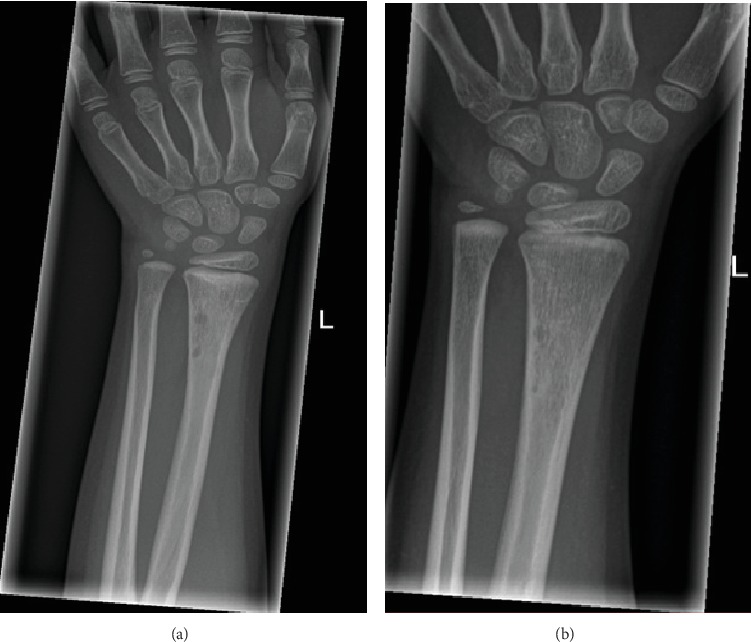
(a) Radiograph showing complete bone healing of the fracture. Two well-circumscribed lucent lesions in the cortex proximal to the previously fractured region. (b) Radiograph showing the lucent lesions fading away.

After four months, she reported for follow-up with X-rays. The images not only showed consolidation of the fracture but also revealed two well-circumscribed lucent lesions in the cortex proximal to the previously fractured region (Figure 4(a)). The lesions were asymptomatic with no signs of inflammation, osteolysis, or osteomyelitis and were diagnosed as benign posttraumatic bone cysts.

She was seen for follow-up once more after six months at which point accompanying X-rays showed a decrease of the lucent lesions (Figure 4(b)). No further follow-up or investigation was required.

## 3. Discussion

Paediatric posttraumatic bone cysts are considered to be a quite rare phenomenon, with only around 30 cases reported in medical journals [1, 2, 7]. They appear in children ranging from 2 to 15 years old and can occur in single form or in multiple lesions. The exact prevalence is unknown; however, it is possible that they have a higher occurrence but remain underreported. Posttraumatic bone cysts usually appear a few months after an initial greenstick fracture [8]. This might add to underreporting, because treatment and radiographic follow-up will often have already ended by then for most children.

Regarding aetiology, the most widely accepted theory is that the lesions develop because of subperiosteal enclosure of intramedullary fat after a fracture [1, 9–11]. Specifically in paediatric patients, the firm periosteum remains intact, allowing extravasated blood and fat derived from the fracture site to be encapsulated underneath. This theory was first suggested by Malghem et al. and supported by their analysis of the cysts on computed tomography (CT), which showed that the lesions seemed to consist of fatty content. They also stated that the lesions might actually appear much sooner after the trauma but initially remain concealed beneath the hematoma on early radiographs. After the hematoma clears up or becomes calcified, the lesions become apparent on radiographs [3].

The cysts are quite rare which is mainly caused by the fact that in order to arise there needs to be a sufficient trauma to cause a disruption of the cortex, while keeping the periosteum intact. In the cases presented, multiple cyst-like lesions are found. Cases of either single or multiple lesions have both been reported; this is probably related to the fracture pattern [1, 2, 7, 8]. The cysts are only reported in children, probably because their periosteum is thicker so it has a higher chance of staying intact during trauma.

While earlier reports mainly focused on imaging and further diagnostics, it is important to note that these benign cysts can easily be recognized on conventional X-rays. This is because of their occurrence after a fracture and their specific location in the cortex, proximal to the fracture. The typical radiological appearance is a radiolucency that is restricted to the cortical bone, having fatty radiodensity and appearing minimally aggressive. The lesions may be multiple or single and are rounded or oval in shape, nonexpansile, and usually less than 1 cm in diameter. Sequential radiographs show the lesions “migrating” away from the epiphyseal disc [4, 10]. Furthermore, these benign cysts eventually resolve spontaneously in 1-3 years, without interfering with the healing process of the fracture [6].

Regarding differential diagnosis, other diagnoses to be considered are simple bone cyst, aneurysmal bone cyst, eosinophilic granuloma, and osteomyelitis. Posttraumatic bone cysts are asymptomatic and are thus not accompanied by fever or pain. When either of these symptoms does occur, the diagnosis becomes less likely and further diagnostic methods are required. In the event of atypical clinical or radiographic presentation, additional imaging using magnetic resonance imaging (MRI) or CT is indicated.

On CT, the lesions can be observed as a fatty radiodensity, possibly accompanied by local thickening of cortical bone [8]. On MRI, the fatty content can be observed as round foci of increased signal intensity on T1-weighted images and intermediate signal intensity on T2-weighted images. Location corresponds to the cystic lesions on radiographs [3].

## 4. Conclusion

Posttraumatic cyst-like lesions are a rare finding after fractures in children. They typically present 2-4 months after the initial trauma as asymptomatic, nonexpansile, well-circumscribed lesions in the cortex. As no treatment is indicated, it is important to recognise them as benign to prevent unnecessary concern and additional diagnostic procedures.

## References

[1] Freire G., Cruz R., Valentim M. H., Maerques T. G., Afonso P. D. (2019). Post-traumatic cyst-like lesion of cortical bone in children. *Skeletal Radiology*.

[2] Houshian S., Pedersen N. W., Torfing T., Venkatram N. (2007). Post-traumatic cortical cysts in paediatric fractures: is it a concern for emergency doctors? A report of three cases. *European Journal of Emergency Medicine*.

[3] Papadimitriou N. G., Christophorides J., Beslikas T. A., Doulianaki E. G., Papadimitriou A. G. (2005). Post-traumatic cystic lesion following fracture of the radius. *Skeletal Radiology*.

[4] Beh J. C. Y., Hamouda E. S. M. (2016). Paediatric post-traumatic osseous cystic lesion following a distal radial fracture. *Journal of Radiology Case Reports*.

[5] Asrian A., Shahabpour M., Tajdar F., de Boeck H. (2010). Posttraumatic cyst-like lesions of cortical bone in children. *Acta Orthopaedica Belgica*.

[6] Pfister-Goedeke L., Braune M. (1981). Cyst-like defects following fractures in children. *Pediatric Radiology*.

[7] Moore T. E., King A. R., Travis R. C., Allen B. C. (1989). Post-traumatic cysts and cyst-like lesions of bone. *Skeletal Radiology*.

[8] Courvoisier A., Bourgeois E., Durand C., Griffet J. (2014). Post-traumatic cyst-like lesion of the radius: a rare but benign lesion. *Diagnostic and Interventional Imaging*.

[9] Malghem J., Maldague B. (1986). Transient fatty cortical defects following fractures in children. *Skeletal Radiology*.

[10] Roach R. T., Cassar-Pullicino V., Summers B. N. (2002). Paediatric post-traumatic cortical defects of the distal radius. *Pediatric Radiology*.

[11] Phillips C. D., Keats T. (1986). The development of post-traumatic cystlike lesions in bone. *Skeletal Radiology*.

